# Dose–Response Evaluation of Dietary Curcumin Supplementation on Ileal Morphology and Antioxidant Status in Broiler Chickens

**DOI:** 10.3390/ani16213337

**Published:** 2026-10-23

**Authors:** Yangyang Hu, Chunmiao Li, Zhaolin Wu, Zhengyang Huang, Zhenhua Zhao

**Affiliations:** Jiangsu Institute of Poultry Science, Yangzhou 225125, China

**Keywords:** broiler, curcumin, intestinal morphology, antioxidant capacity, Nrf2 signaling pathway

## Abstract

This study aimed to evaluate suitable dietary curcumin supplementation levels for improving intestinal morphology and antioxidant status in medium-growing broiler chickens fed a practical corn–soybean meal diet from 57 to 84 d of age. A total of 252 male broilers (57 d of age; 1092.86 ± 8.91 g) were randomly assigned to six dietary treatments with seven replicate cages of six birds each. Broilers were fed diets supplemented with 0, 80, 160, 240, 320, or 400 mg/kg curcumin for 28 d. Asymptotic, broken-line, and quadratic models were fitted to dose–response data to estimate suitable dietary curcumin supplementation levels. Curcumin supplementation improved ileal morphology and antioxidant status, as evidenced by increased villus height (VH), VH/crypt depth (CD), villus surface area, total antioxidant capacity, and superoxide dismutase (SOD) activity, together with decreased CD and malondialdehyde (MDA) content. Curcumin also upregulated the mRNA expression of catalase (*CAT*), *SOD*, glutathione peroxidase (*GPX*)*1*, *GPX3*, *GPX4*, and nuclear factor erythroid 2-related factor 2 (*Nrf2*) and increased Nrf2 protein abundance. Among the candidate models, broken-line regression provided the best fit for key ileal antioxidant indices, indicating that approximately 188 mg/kg of dietary curcumin may be sufficient to maximize ileal total antioxidant capacity under the present experimental conditions. Furthermore, Nrf2 expression exhibited trends similar to those of antioxidant-related gene expression and antioxidant enzyme activities, but showed an opposite trend to MDA content. These findings suggest a potential association between Nrf2 expression and intestinal antioxidant status.

## Simple Summary

Curcumin is a natural plant-derived compound known for its antioxidant activity. In this study, we investigated the effects of dietary curcumin supplementation from 57 to 84 d of age on intestinal health in broilers. The results showed that curcumin improved ileal structure and enhanced the antioxidant defense capacity of broilers. These beneficial effects were accompanied by changes in antioxidant-related gene expression and Nrf2 mRNA and protein levels. Under the present experimental conditions, dietary supplementation with approximately 188 mg/kg of curcumin may be recommended to maximize the total antioxidant capacity of the small intestine in broilers. These findings suggest that curcumin may serve as a potential natural feed additive to improve intestinal health and oxidative balance in broilers.

## 1. Introduction

Since the ban on antibiotic growth promoters in animal feed, natural and health-promoting feed additives have received increasing attention in the global livestock industry [1]. Curcumin, a bioactive polyphenolic compound derived from turmeric, possesses a range of biological activities, including anti-inflammatory, antioxidant, antiviral, and antibacterial effects [2]. Previous studies in broilers have shown that dietary curcumin supplementation can improve growth performance [3], modulate gut microbiota [4], enhance immune function, maintain intestinal integrity and barrier function [3], and improve meat quality [5]. Intestinal health is an important determinant of physiological homeostasis and productive performance in poultry, and an adequate antioxidant defense system is essential for maintaining intestinal structural and functional integrity [6,7,8]. Curcumin nanoparticles have also been reported to improve intestinal health in broilers, as evidenced by increased small intestinal villus height (VH), VH/crypt depth (CD), and the activities of glutathione peroxidase (GPX) and superoxide dismutase (SOD), along with reduced villus width, CD, and malondialdehyde (MDA) levels [9]. However, although the beneficial effects of dietary curcumin have been widely reported, the dose–response relationship between curcumin supplementation and intestinal antioxidant responses has not been systematically characterized, and a suitable supplementation level based on intestinal antioxidant criteria remains to be estimated.

Intestinal redox homeostasis depends largely on endogenous antioxidant defense systems, particularly antioxidant enzymes that protect intestinal tissues against oxidative damage. Nuclear factor erythroid 2-related factor 2 (Nrf2) is an important transcriptional regulator involved in the expression of antioxidant enzymes and cytoprotective genes [10]. Previous studies have suggested that dietary curcumin may enhance antioxidant enzyme expression and improve small intestinal morphology, with these effects potentially associated with Nrf2-related antioxidant responses [10,11]. Nevertheless, inconsistent responses to dietary curcumin supplementation have been reported for growth performance, intestinal morphology, antioxidant status, and meat quality in broilers [12,13,14,15]. These discrepancies may partly reflect differences in supplementation level. However, the dose-dependent relationship between dietary curcumin and intestinal antioxidant responses, particularly Nrf2-related antioxidant regulation, remains poorly understood.

The crossbred black-bone broilers used in the present study were derived from Lueyang black-bone chickens as the maternal line and Guangxi Lingshan indigenous black-feathered chickens as the paternal line. These crossbred birds combine the characteristic pigmentation of black-bone chickens with relatively favorable growth performance and feed efficiency and have been increasingly used in commercial production. Maintaining intestinal health is therefore important for supporting their productive potential. We hypothesized that supplementation with an appropriate level of dietary curcumin would improve intestinal morphology and antioxidant capacity, with these improvements being associated with enhanced Nrf2-related antioxidant responses. Accordingly, the present study aimed to evaluate the dose–response effects of graded dietary curcumin supplementation on ileal morphology, antioxidant-related enzymes, oxidative products, antioxidant-related gene expression, and Nrf2-related antioxidant responses in broilers, and to estimate a suitable supplemental level of curcumin using dose–response models.

## 2. Materials and Methods

### 2.1. Animals, Feed Preparation and Experimental Design

A total of 350 one-day-old male black-bone broilers from a commercial crossed line (Lueyang black-bone chicken × Guangxi Lingshan indigenous black-feathered chicken) were obtained from the Jiangsu Institute of Poultry Science (Yangzhou, China). The resulting crossbred offspring are medium-growing commercial broilers. From 1 to 56 d of age, all birds were fed a corn–soybean meal basal diet. At 57 d of age, 252 broilers with similar body weights were selected and randomly assigned to six dietary treatments, each consisting of seven replicate cages with six birds per cage. Each cage was regarded as one replicate unit. Broilers had ad libitum access to feed and water throughout the 28 d experimental period. Birds were housed under a lighting schedule of 18 h light and 6 h darkness (18L:6D) in a completely randomized design. Body weight was recorded weekly throughout the experiment, and the corresponding growth-performance data have been included in a separate manuscript submitted to another journal (Hu et al., submitted). All experimental procedures were approved by the Animal Care and Use Committee of the Institute of Poultry Science, Jiangsu Province (approval code: CAS-JIPS250912; approval date: 12 September 2025). Six experimental diets were formulated by supplementing the basal diet with 0, 80, 160, 240, 320, or 400 mg/kg curcumin. The commercial curcumin preparation used in the present study was provided by Kehu Biotechnology Research Center (Guangzhou, China) and had a purity of 92%. The dietary supplementation levels were selected according to previous studies [3,16]. The basal diet was formulated to meet or exceed the nutrient requirements recommended by the NRC [17], the Feeding Standard of Chicken in China [18], and the Nutrient Requirements of Yellow-feathered Broiler Chickens (NY/T 3645-2020) [19]. The ingredient composition and nutrient levels of the basal diet are presented in Table 1.

### 2.2. Sample Collections and Preparations

Diet samples from each treatment group were collected using the quartering method for the determination of calcium and crude protein contents. At 84 d of age, all broilers were fasted overnight with free access to water. Three broilers per replicate with body weights close to the replicate mean were selected for sample collection. The selected birds were anesthetized by intraperitoneal injection of propofol (20 mg/kg body weight) and subsequently euthanized by cervical dislocation. A 2 cm segment from the mid-ileum of one bird was excised and fixed in 4% paraformaldehyde for subsequent histomorphological analysis. The remaining ileum from this bird and the entire ilea from the other two birds were flushed with ice-cold physiological saline to remove luminal contents. The intestinal mucosa was then gently scraped using a sterile glass slide, immediately transferred into sterile 1.5 mL centrifuge tubes, snap-frozen in liquid nitrogen, and stored at −80 °C until subsequent analyses of enzyme activities and gene expression. For samples collected from three chickens, equal proportions of the individual samples were pooled to generate a single composite sample for subsequent analysis.

### 2.3. Analysis of Dietary Crude Protein and Calcium Content and Ileal Oxidative Stress Indicators Level

We used the Kjeldahl method [20] to determine crude protein and an ICP-OES (5110 ICP-OES, Agilent Technologies Australia (M) Pty Ltd., Mulgrave, Australia) to determine calcium content in the diets. Commercial kits were used to determine the contents of MDA (cat no: A003-1-2; Nanjing Jiancheng, Nanjing, China) and total antioxidant capacity (T-AOC) (cat no: A015-2-1; Nanjing Jiancheng, Nanjing, China), as well as the activity of SOD (cat no: A001-2-2; Nanjing Jiancheng, Nanjing, China) in the ileum.

### 2.4. Histological Morphology Assessment of the Ileum

The histological morphology of the ileum was assessed following a previously described method [21]. Three of the seven replicate cages per treatment were randomly selected for histomorphological analysis. Briefly, formalin-fixed intestinal samples were embedded in paraffin, and serial sections (5 µm thick) were stained with hematoxylin and eosin (H&E) for histomorphological observation. For each sample, VH and CD were measured in 10 randomly selected villi–crypt units, and the VH/CD ratio was subsequently calculated. The 10 subsample measurements were averaged for each biological sample before statistical analysis. Villus surface area (VSA) was calculated using the formula VSA = 2π × VH × (villus width/2), where villus width was defined as the distance between the bases of the villi (the villus–intestinal gland junction). All histomorphological examinations and measurements were performed using an Olympus optical microscope and Mingmei image-analysis software (version 9; Guangzhou, China).

### 2.5. RT-qPCR

Total RNA was isolated from approximately 50 mg of ileal tissue using an RNA Tissue Kit (cat no: R401-01; Vazyme, Nanjing, China). The isolated RNA was reverse-transcribed into cDNA using a commercial reverse transcription kit (cat no: R223-01; Vazyme, Nanjing, China). Subsequently, SYBR Green-based quantitative real-time PCR was performed using the Q111-02 reagent (Vazyme, Nanjing, China). All primers were synthesized by Tsingke Biotechnology, and their sequences are listed in Table 2. Relative gene expression levels were calculated using the 2^−ΔΔCT^ method [22], with β-actin used as the internal reference gene [23].

### 2.6. Western Blot

Frozen ileal mucosa samples were homogenized in ice-cold RIPA lysis buffer (cat no: R0010; Solarbio, Beijing, China) as previously described [24]. Protein concentrations were determined using a BCA protein assay kit (cat no: 23225, Thermo Fisher Scientific, Waltham, MA, USA). Equal amounts of protein (40 μg) were separated by SDS-PAGE and transferred onto nitrocellulose (NC) membranes. The membranes were blocked with 5% BSA for 1.5 h at room temperature and subsequently washed with Tris-buffered saline containing Tween 20 (TBST). The membranes were then incubated overnight at 4 °C with rabbit anti-Nrf2 (cat no: WL02135; Wanlei, Shenyang, China, 1:1000) [25] and mouse anti-β-actin (cat no: HX1827; Huaxingbio, Beijing, China, 1:5000). After washing with TBST, the membranes were incubated with the appropriate secondary antibodies (anti-mouse IgG, cat no: HX2032; anti-rabbit IgG, cat no: HX2031; Huaxingbio, Beijing, China, 1:5000). Protein bands were visualized using an enhanced chemiluminescence (ECL) detection kit (cat no: 180-5001; Tanon, Shanghai, China). Band intensities were quantified using ImageJ software (version 1.6.0), and Nrf2 protein expression was normalized to β-actin. Quantitative analysis was performed using seven independent biological replicates per treatment, with one representative sample from each treatment group displayed in the figure.

### 2.7. Statistical Analyses

Except for correlation analysis, all statistical analyses were performed using SAS software (version 9.4, 2013). Each cage was considered an experimental unit, with seven replicate cages per treatment. For ileal histomorphological parameters, three replicates per treatment were randomly selected for analysis, whereas seven replicates per treatment were used for all other parameters. For all measured parameters, observations falling outside the mean ± 2 standard deviations (SDs) were identified as outliers and excluded from statistical analysis. Consequently, the final number of replicates varied among parameters, with the actual sample sizes indicated in the corresponding tables and figure legends. Prior to one-way analysis of variance (ANOVA), the normality of residuals and homogeneity of variances were assessed using the Shapiro–Wilk test and Levene’s test, respectively. Data from different treatment groups were analyzed using one-way ANOVA with the GLM procedure, and differences among means were compared using the least significant difference (LSD) method. Pearson’s correlation analysis was performed using individual biological replicate data from six treatment groups using the Hiplot platform (https://hiplot.com.cn; accessed on 15 October 2026). The effective sample size varied among correlations due to the exclusion of individual observations. No adjustment for multiple comparisons was applied. Orthogonal contrast analysis was employed to evaluate the linear and quadratic responses of selected parameters to increasing levels of dietary curcumin supplementation, with contrast coefficients calculated based on the actual supplementation level. Orthogonal contrasts were used to assess dose-dependent trends, whereas asymptotic, broken-line, and quadratic models were fitted to estimate potentially suitable dietary curcumin supplementation levels. Furthermore, several mathematical models, including asymptotic, broken-line, and quadratic models, were fitted to representative data to estimate potentially suitable dietary curcumin supplementation levels for broilers. The appropriate levels were defined as the dose required to achieve 95% of the maximum response for the asymptotic model, the breakpoint for the broken-line model, and the maximum response point for the quadratic model [24,26,27]. The statistical significance was set at *p* < 0.05.

## 3. Results

### 3.1. Ileal Histological Morphology

**Figure 1:**
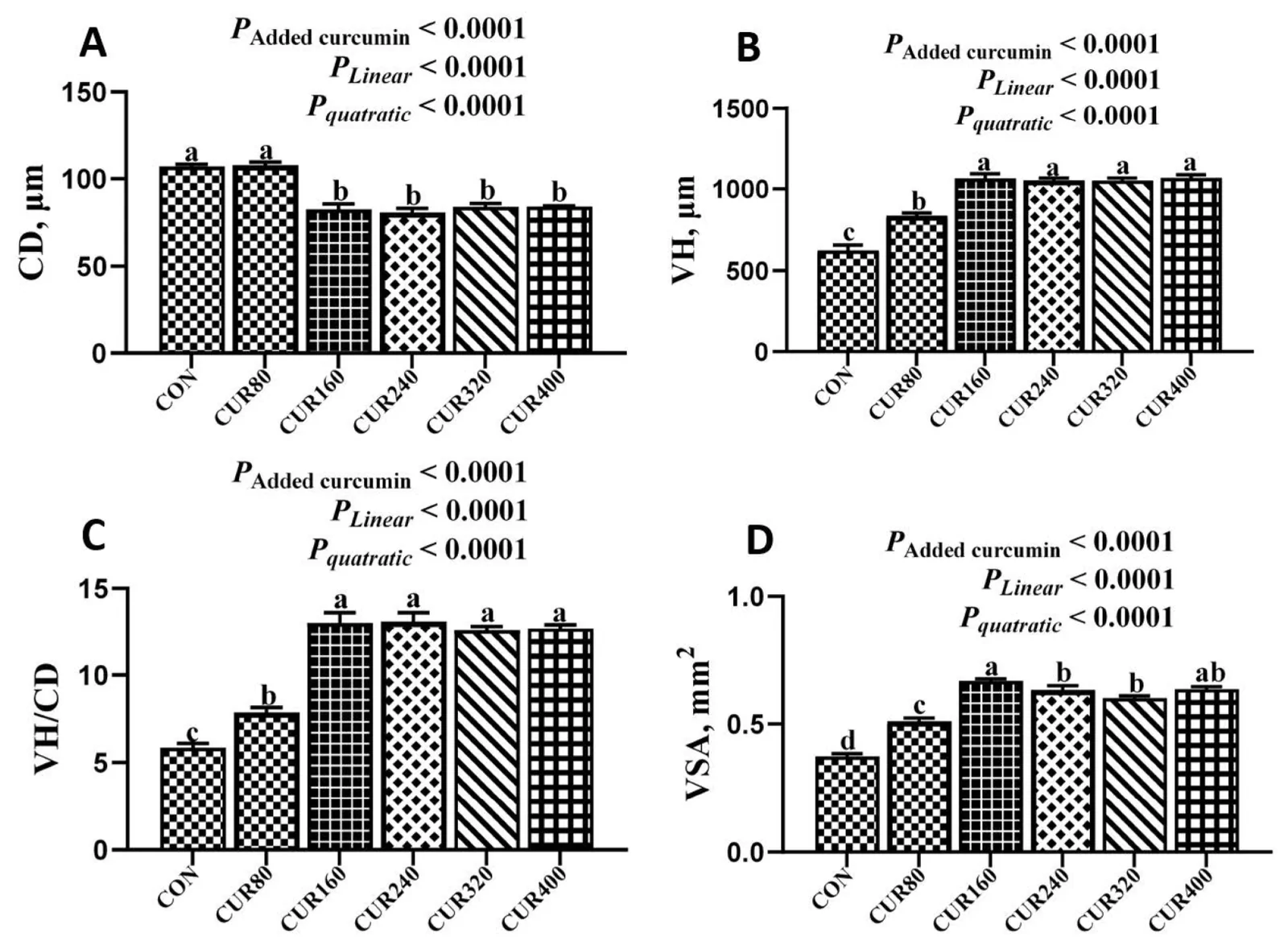
Effects of graded levels of dietary curcumin on histological morphology of the ileum in broilers at 84 d of age. (**A**) CD. (**B**) VH. (**C**) VH/CD. (**D**) VSA. CD: crypt depth. VH: villus height. VH/CD: villus height/crypt depth. VSA: villus surface area. CON: control group. CUR80: add 80 mg/kg curcumin to the basal diet. CUR160: add 160 mg/kg curcumin to the basal diet. CUR240: add 240 mg/kg curcumin to the basal diet. CUR320: add 320 mg/kg curcumin to the basal diet. CUR400: add 400 mg/kg curcumin to the basal diet. Different letters indicate significant differences among treatments (*p* < 0.05). Data are means ± SE (*n* = 3).

As shown in Figure 1, dietary curcumin supplementation significantly affected (*p* < 0.0001) ileal CD, VH, VH/CD, and VSA. As the dietary curcumin level increased, CD decreased significantly (*p* < 0.05) with increasing dietary curcumin levels up to 160 mg/kg, after which it remained relatively stable. In contrast, VH and VH/CD increased significantly (*p* < 0.05), reaching their highest values at 160 mg/kg, with no additional improvement at higher supplementation levels. Similarly, VSA increased significantly (*p* < 0.05) with increasing dietary curcumin levels, reached its maximum value at 160 mg/kg, and then declined significantly (*p* < 0.05) with further increases in curcumin supplementation. Furthermore, dietary curcumin supplementation exerted significant linear and quadratic effects on all intestinal morphological parameters (*p* < 0.0001). Specifically, VH and VH/CD increased linearly and quadratically, whereas CD decreased linearly and quadratically. Likewise, VSA exhibited significant linear and quadratic responses to increasing dietary curcumin levels.

### 3.2. Ileal T-AOC and MDA Contents as Well as SOD Activity

**Figure 2:**
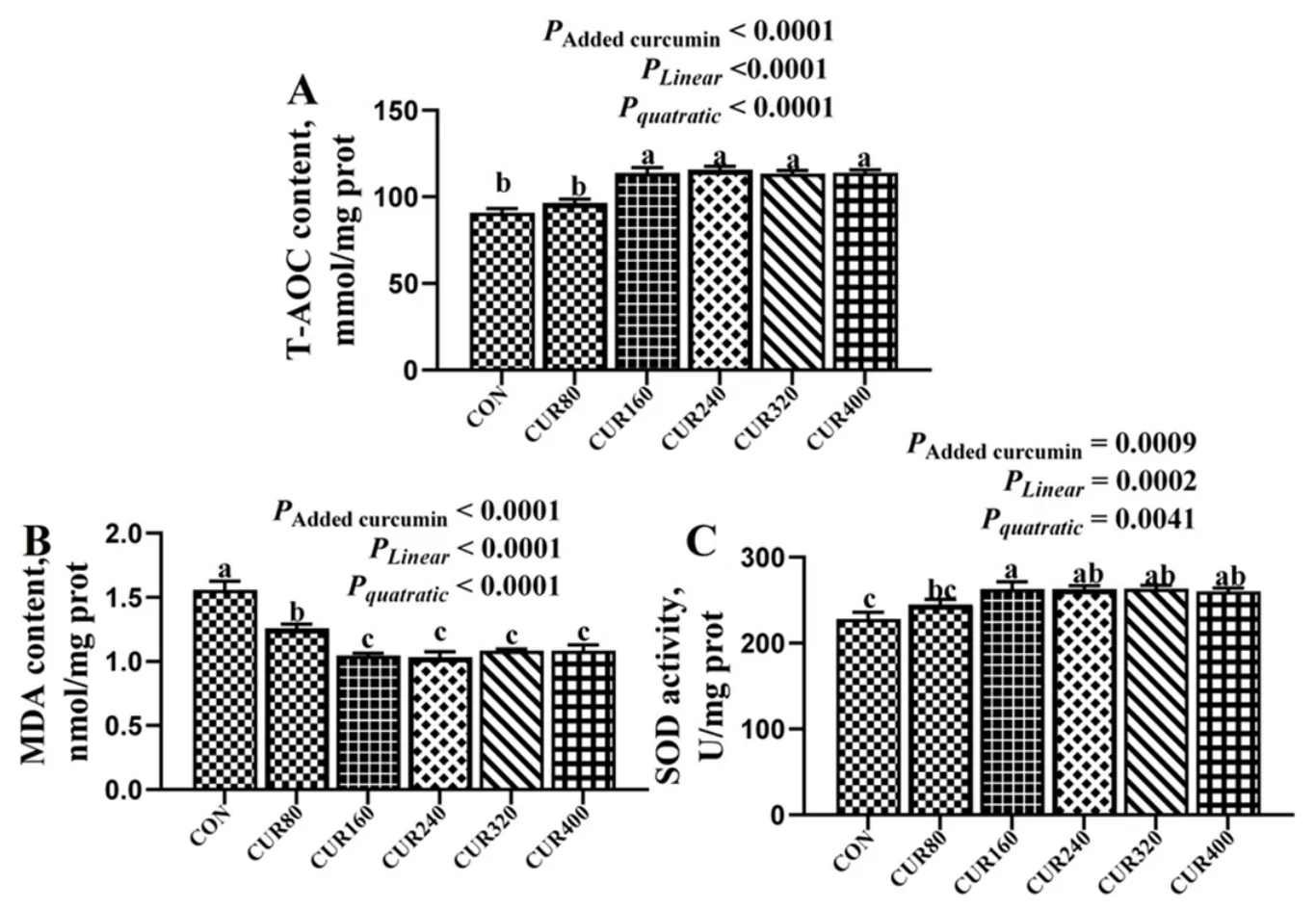
Effects of graded levels of dietary curcumin on antioxidant enzyme and peroxide product levels of the ileum in broilers at 84 d of age. (**A**) T-AOC content. (**B**) MDA content. (**C**) SOD activity. T-AOC: total antioxidant capacity. MDA: malondialdehyde. SOD: superoxide dismutase. CON: control group. CUR80: add 80 mg/kg curcumin to the basal diet. CUR160: add 160 mg/kg curcumin to the basal diet. CUR240: add 240 mg/kg curcumin to the basal diet. CUR320: add 320 mg/kg curcumin to the basal diet. CUR400: add 400 mg/kg curcumin to the basal diet. Different letters indicate significant differences among treatments (*p* < 0.05). Data are means ± SE (*n* = 5–6).

As shown in Figure 2, dietary curcumin supplementation significantly influenced (*p* < 0.01) ileal T-AOC, MDA content, and SOD activity. Ileal T-AOC content and SOD activity increased significantly (*p* < 0.05) with increasing dietary curcumin supplementation, reaching maximal values at 160 mg/kg, beyond which no further improvement was observed. In contrast, ileal MDA content decreased significantly (*p* < 0.05), reaching its minimum at 160 mg/kg and remaining unchanged at higher supplementation levels. Furthermore, ileal T-AOC content and SOD activity exhibited significant linear and quadratic increases (*p* < 0.01), whereas ileal MDA content showed significant linear and quadratic decreases (*p* < 0.0001) in response to increasing dietary curcumin supplementation.

### 3.3. mRNA Expression Levels

**Figure 3:**
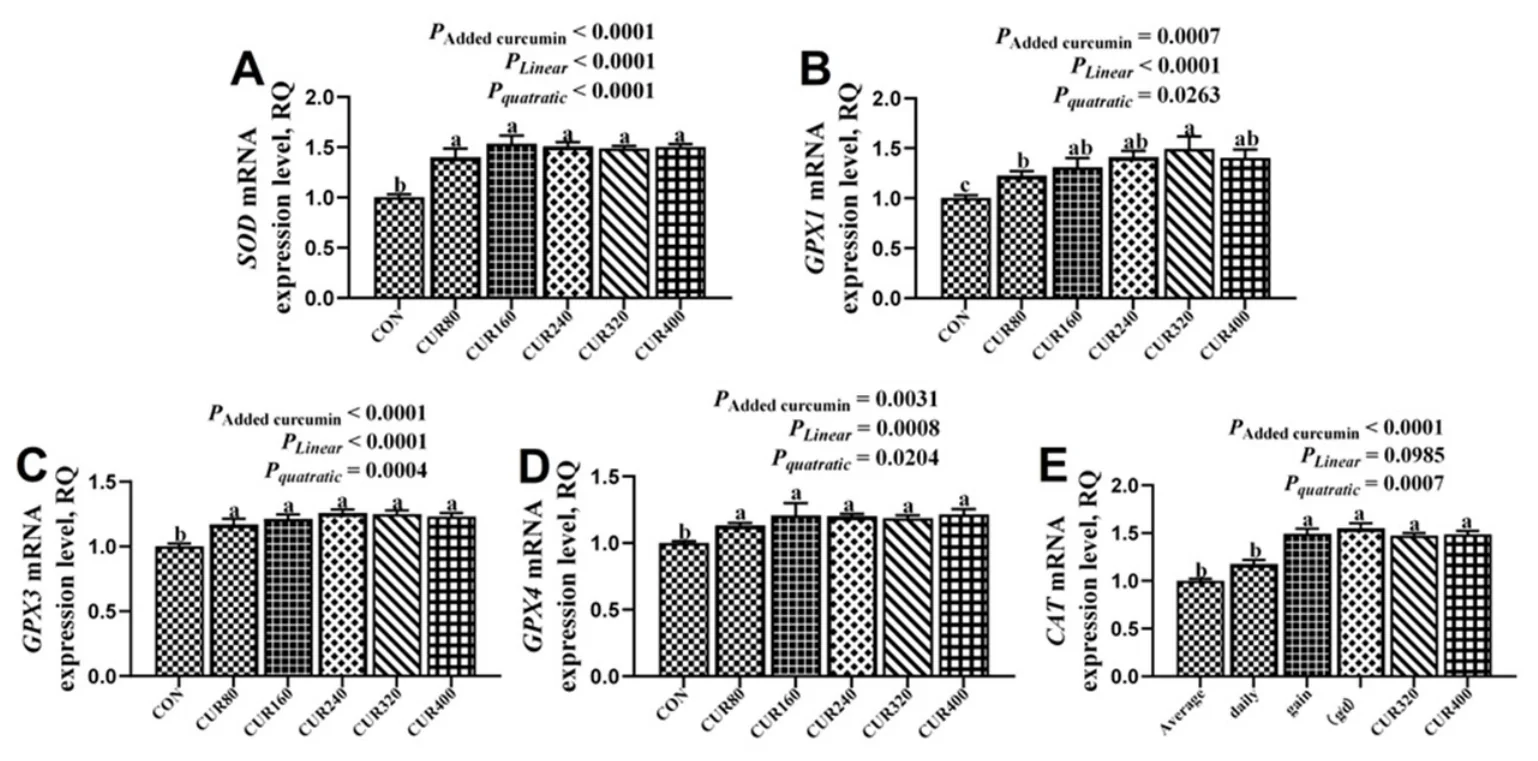
Effects of graded levels of dietary curcumin on the mRNA expression levels of antioxidant-related genes in the ileum of 84 d old broilers. (**A**) mRNA expression level of *SOD*. (**B**) mRNA expression level of *GPX1*. (**C**) mRNA expression level of *GPX3*. (**D**) mRNA expression level of *GPX4*. (**E**) mRNA expression level of *CAT*. CAT: catalase. SOD: superoxide dismutase. GPX: glutathione peroxidase. Values of mRNA abundance levels of target genes were calculated as the relative quantities (RQ) of *CAT*, *SOD*, *GPX1*, *GPX3* or *GPX4* mRNA to the internal reference gene β-actin mRNA using 2^−△△Ct^. CON: control group. CUR80: add 80 mg/kg curcumin to the basal diet. CUR160: add 160 mg/kg curcumin to the basal diet. CUR240: add 240 mg/kg curcumin to the basal diet. CUR320: add 320 mg/kg curcumin to the basal diet. CUR400: add 400 mg/kg curcumin to the basal diet. Different letters indicate significant differences among treatments (*p* < 0.05). Data are means ± SE (*n* = 5–7).

**Figure 4:**
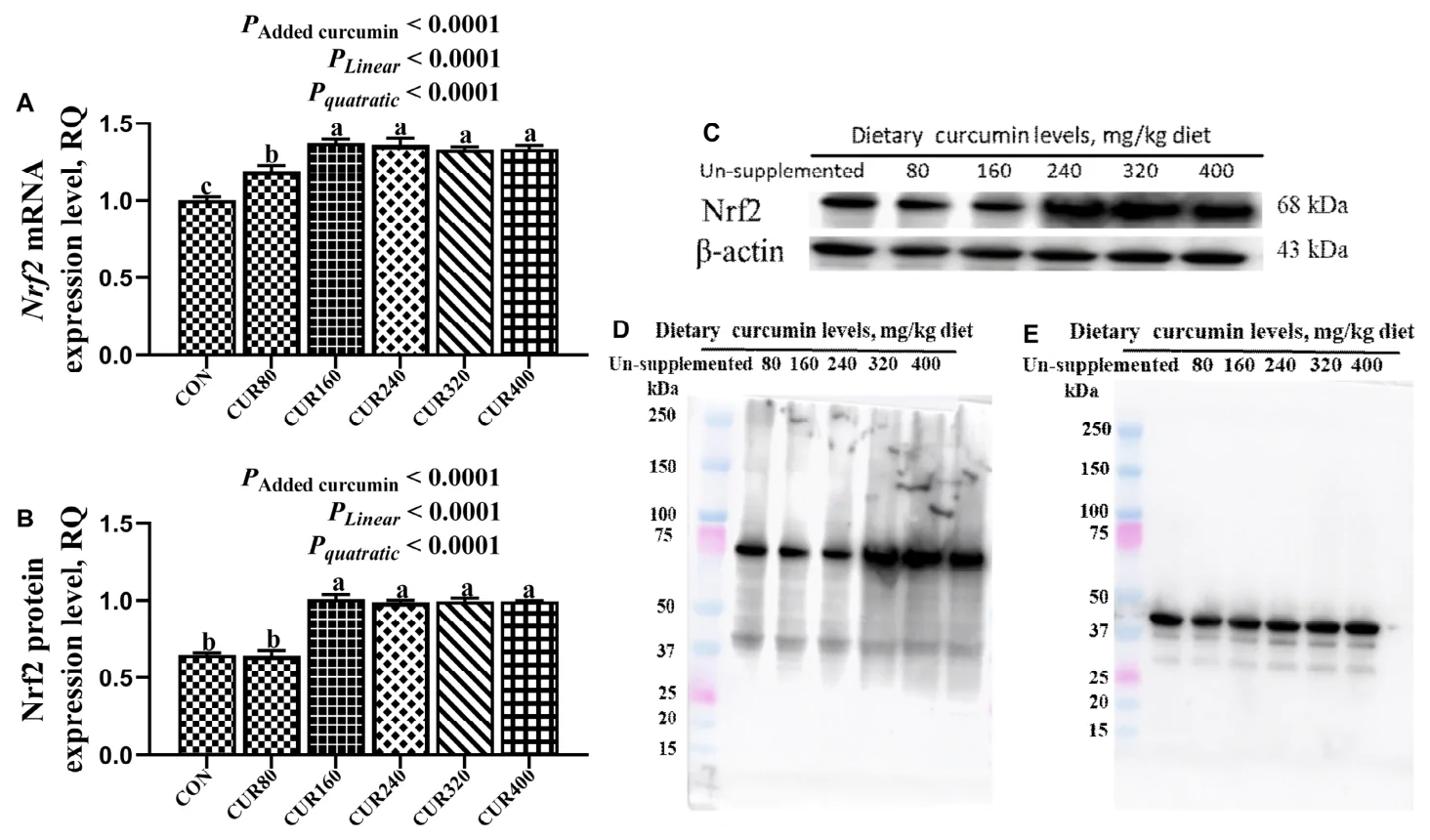
Effects of graded levels of dietary curcumin on the mRNA or protein expression levels of Nrf2 in the ileum of 84 d old broilers. (**A**) mRNA expression level of *Nrf2*. (**B**) Protein expression level of Nrf2. (**C**) Representative immunoblot of Nrf2. (**D**) Full-length immunoblot of Nrf2. (**E**) Full-length immunoblot of β-actin. Nrf2: nuclear factor erythroid 2-related factor 2. Value of mRNA abundance level was calculated as the relative quantification (RQ) of *Nrf2* mRNA to the internal reference gene β-actin mRNA using 2^−△△Ct^. Value of Nrf2 protein expression level was expressed by the relative quantification (RQ) of Nrf2 protein band intensity to the internal reference band intensity of β-actin. Different letters indicate significant differences among treatments (*p* < 0.05). Data are means ± SE (*n* = 6).

As shown in Figure 3 and Figure 4A, dietary curcumin supplementation significantly influenced (*p* < 0.02) the expression of antioxidant-related genes in the ileal mucosa. The mRNA expression levels of *SOD*, *GPX3*, and *GPX4* increased significantly (*p* < 0.05) in response to increasing dietary curcumin supplementation, reaching maximal values at 80 mg/kg, beyond which no further increases were detected. Similarly, *CAT* and *Nrf2* mRNA expression increased significantly (*p* < 0.05), peaking at 160 mg/kg and remaining stable thereafter. *GPX1* mRNA expression also increased significantly (*p* < 0.05), reaching its maximum at 320 mg/kg, with no additional increase observed at higher supplementation levels.

Additionally, the mRNA expression levels of *SOD*, *GPX1*, *GPX3*, *GPX4*, and *Nrf2* increased linearly and quadratically (*p* < 0.03) with increasing dietary curcumin supplementation, whereas *CAT* mRNA expression exhibited a significant quadratic response (*p* < 0.01).

### 3.4. Protein Expression Level

As shown in Figure 4B, dietary curcumin supplementation significantly influenced (*p* < 0.0001) the protein expression level of Nrf2 in the ileal mucosa. The protein expression of Nrf2 increased significantly (*p* < 0.0001) with increasing dietary curcumin supplementation, reaching a maximum at 160 mg/kg and remaining relatively stable at higher supplementation levels. Moreover, Nrf2 protein expression exhibited significant linear and quadratic responses to increasing dietary curcumin supplementation (*p* < 0.0001).

### 3.5. Correlation Analysis

**Figure 5:**
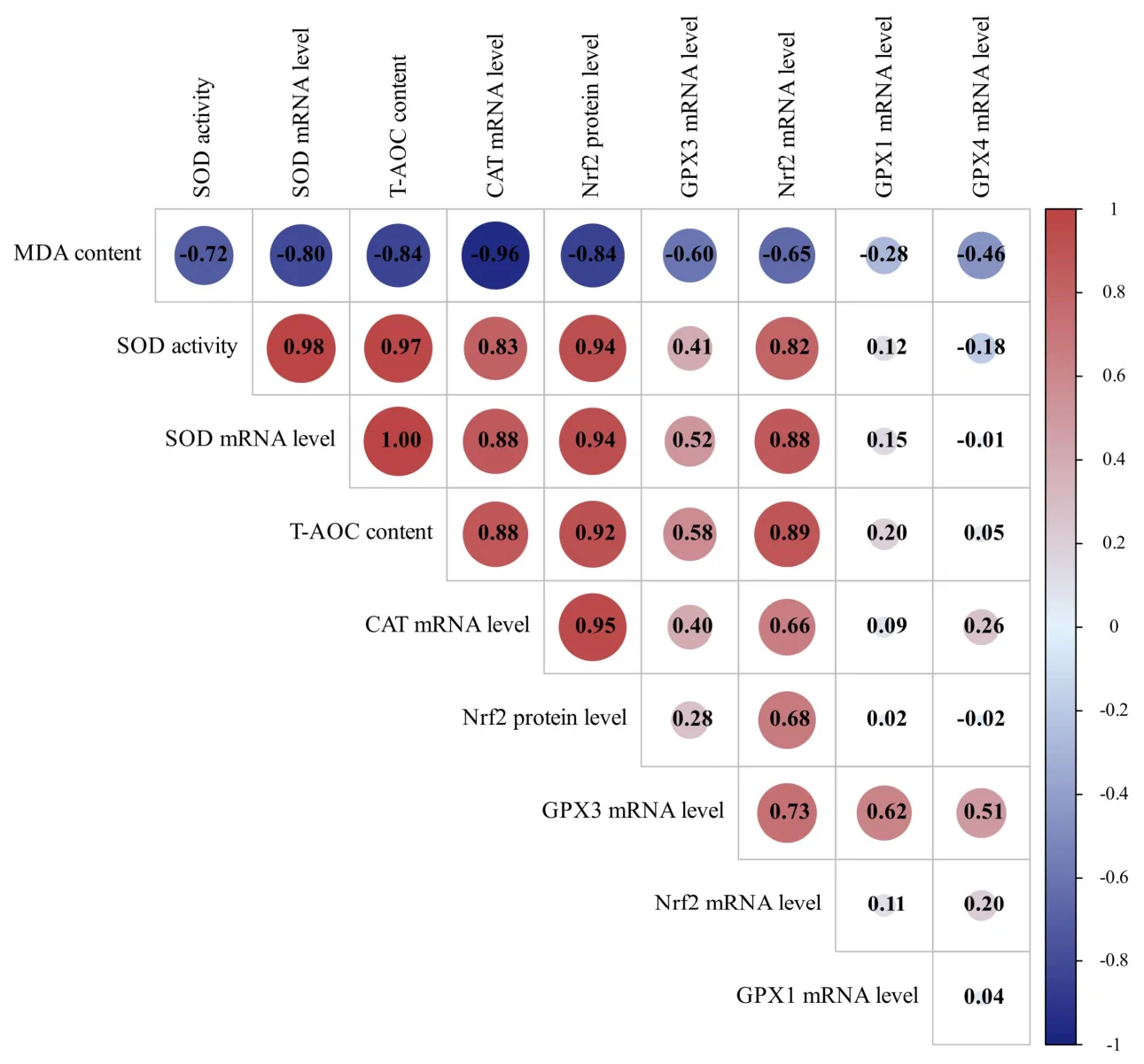
Correlation analysis of the antioxidant-related substances, genes, and related signaling molecule in the ileum of 84 d old broilers. CAT: catalase. SOD: superoxide dismutase. GPX: glutathione peroxidase. Nrf2: nuclear factor erythroid 2-related factor 2. T-AOC: total antioxidant capacity. MDA: malondialdehyde. Red represents positive correlation and blue represents negative correlation; the darker the color, the stronger the correlation. Pearson’s correlation analysis was performed using individual biological replicate data (5–7 replicates per treatment).

As shown in Figure 5, correlation heatmaps were generated using the Hiplot tool (https://hiplot.com.cn) to visualize the patterns of variation among antioxidant-related indices, gene expression, and related signaling molecules across dietary curcumin treatments. Most antioxidant-related indices and antioxidant-related gene expression levels exhibited similar variation patterns to those of ileal Nrf2 mRNA and protein expression. In contrast, ileal MDA content showed an opposite pattern.

### 3.6. Estimation of Suitable Dietary Curcumin Supplementation Levels

**Figure 6:**
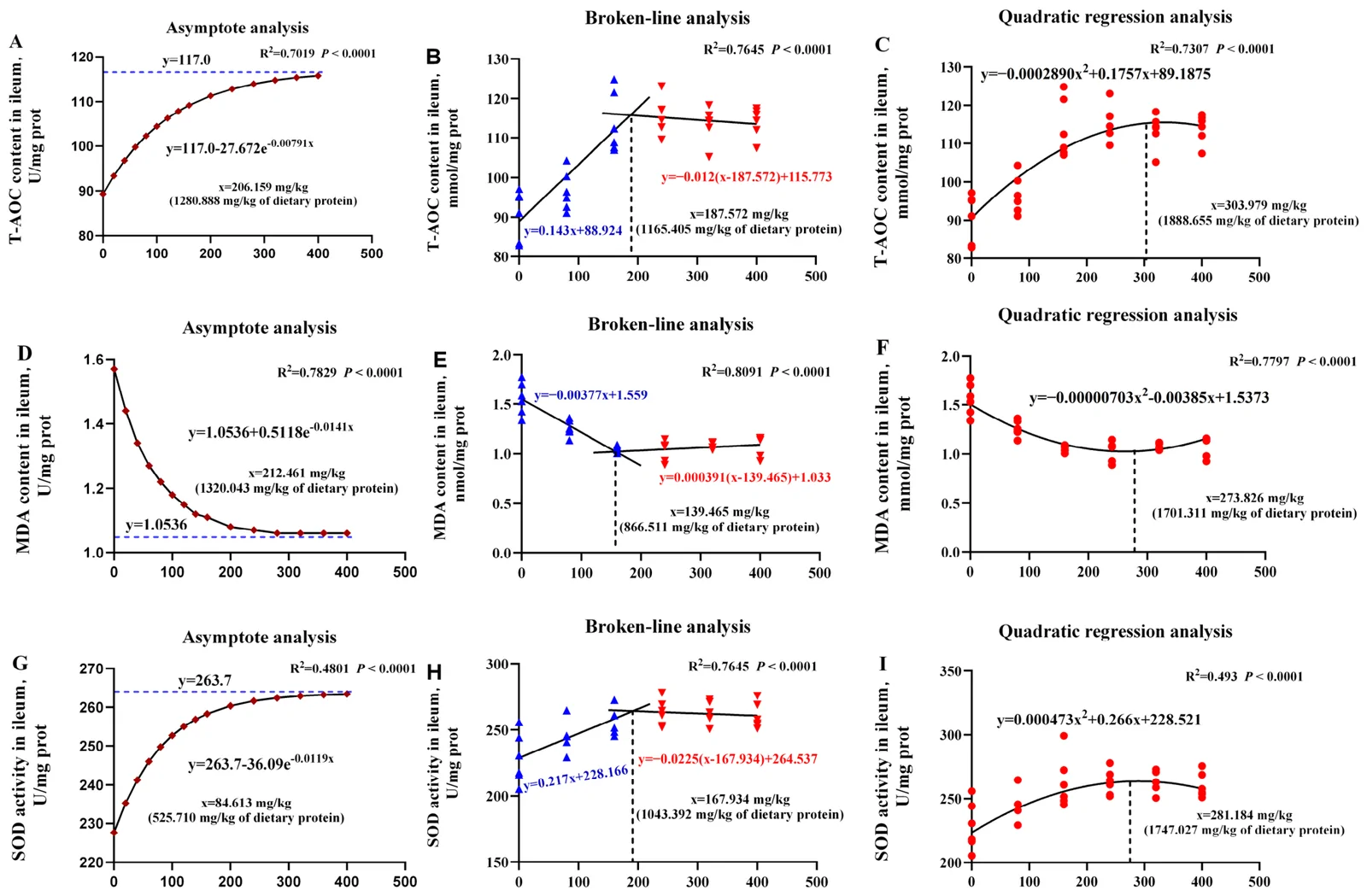
Model-based estimates of dietary curcumin supplementation levels for broilers from 57 to 84 d of age using asymptotic, broken-line, and quadratic models. (**A**) Asymptotic analysis of T-AOC content in the ileum. (**B**) Broken-line analysis of T-AOC content in the ileum. (**C**) Quadratic analysis of T-AOC content in the ileum. (**D**) Asymptotic analysis of MDA content in the ileum. (**E**) Broken-line analysis of MDA content in the ileum. (**F**) Quadratic analysis of MDA content in the ileum. (**G**) Asymptotic analysis of SOD activity in the ileum. (**H**) Broken-line analysis of SOD activity in the ileum. (**I**) Quadratic analysis of SOD activity in the ileum. T-AOC: total antioxidant capacity. MDA: malondialdehyde. SOD: superoxide dismutase. The regression equation was fitted according to dietary added curcumin levels (0, 80, 160, 240, 320 and 400 mg/kg), where Y was the measured index and X was dietary curcumin added level (mg/kg) in the basal diet.

As shown in Figure 6, the dietary curcumin supplementation levels for medium-growing broilers from 57 to 84 d of age were estimated using three regression models. Based on the asymptotic model, the supplementation levels derived from T-AOC, MDA, and SOD were 206.159, 212.461, and 84.613 mg/kg diet, corresponding to 1280.888, 1320.043, and 525.710 mg/kg dietary protein, respectively. Using the broken-line model, the corresponding estimates were 187.572, 139.465, and 167.934 mg/kg diet, equivalent to 1165.405, 866.511, and 1043.392 mg/kg dietary protein, respectively. Based on the quadratic model, the estimated supplementation levels were 303.979, 273.826, and 281.184 mg/kg diet, corresponding to 1888.655, 1701.311, and 1747.027 mg/kg dietary protein for T-AOC, MDA, and SOD, respectively.

## 4. Discussion

Since July 2020, China has completely banned the use of antibiotics in animal feed, highlighting the urgent need for safe and residue-free alternatives. Plant-derived feed additives have attracted considerable attention because of their low toxicity and broad biological activities [28]. Curcumin, a natural polyphenolic compound derived from turmeric, possesses anti-inflammatory, antioxidant, antiviral and antibacterial properties [29,30,31]. Previous studies have demonstrated that dietary curcumin supplementation improves the health and productive performance of broilers [32]. Intestinal health is fundamental to nutrient utilization and overall physiological function. Intestinal morphology is widely recognized as an important indicator of intestinal health, with CD reflecting epithelial cell turnover and VH, VSA, and the VH/CD ratio indicating intestinal absorptive capacity [33,34,35]. Consistent with previous studies [5,16], dietary curcumin supplementation in the present study increased VH, VSA, and the VH/CD ratio while reducing CD, suggesting an improvement in intestinal morphology. However, inconsistent responses have also been reported. For example, Zhang et al. [10] reported that dietary curcumin supplementation significantly increased ileal CD but had no significant effects on ileal CAT and SOD activities and MDA content. Similarly, Guo et al. [36] found that dietary curcumin supplementation had no significant effects on ileal T-AOC and MDA contents or SOD and CAT activities in Ross 308 broilers. These discrepancies may be attributed to differences in broiler genotype, age, dietary composition, curcumin dosage, and experimental duration.

Alongside these morphological benefits, intestinal health also relies on an effective antioxidant defense system. T-AOC reflects the overall antioxidant capacity of tissues, whereas MDA is a widely accepted marker of lipid peroxidation [5,37]. Consistent with previous reports [38,39], dietary curcumin supplementation increased ileal T-AOC and SOD activity while reducing MDA content. In addition, dietary curcumin upregulated the mRNA expression of several antioxidant-related genes, including *SOD*, *CAT*, *GPX1*, *GPX3*, and *GPX4*, further supporting the improvement of intestinal antioxidant defense. Curcumin supplementation also increased ileal Nrf2 mRNA and protein expression, which exhibited similar response patterns to antioxidant enzyme expression but opposite patterns to MDA content across dietary curcumin treatments. Together with previous findings, these results suggest that curcumin-induced antioxidant regulation may be associated with Nrf2-related signaling. Although previous dose–response studies and meta-analyses have reported the beneficial effects of dietary curcumin supplementation in broilers, the present study extends these findings by specifically examining medium-growing broilers aged 57–84 d. By integrating assessments of ileal morphology, antioxidant status, and Nrf2 protein expression, this study provides a more comprehensive evaluation of intestinal responses to dietary curcumin. Furthermore, the comparison of three regression models based on ileal T-AOC, MDA content, and SOD activity provides an age- and diet-specific estimate of the appropriate curcumin supplementation range.

In the present study, significant linear and quadratic dose–response relationships were observed between dietary curcumin supplementation and ileal morphology, antioxidant enzyme activities, oxidative status, and antioxidant-related gene expression (except *CAT* mRNA expression, which showed only a quadratic response). Given that small intestinal antioxidant enzyme activities and lipid peroxidation products are important indicators of intestinal oxidative status [40], three regression models were further compared to estimate the appropriate curcumin supplementation level based on ileal T-AOC, MDA content, and SOD activity. Among the three models evaluated, the broken-line model exhibited the highest R^2^ values for T-AOC, MDA content, and SOD activity, indicating a better fit to the observed dose–response relationships. Based on this model, the estimated curcumin supplementation levels derived from these three antioxidant parameters ranged from 139.465 to 187.572 mg/kg in a practical corn–soybean meal-based diet for broilers aged 57–84 d. This range was comparable to the effective supplementation levels reported in previous studies on broilers [41,42]. The estimated breakpoints suggest that the antioxidant responses to dietary curcumin supplementation may approach a plateau at higher supplementation levels, beyond which further increases may provide limited additional benefits. Consistent with this interpretation, Yadav et al. [3] reported that glutathione and total glutathione levels tended to stabilize at higher curcumin supplementation levels, indicating a plateau in the dose–response relationship. Similarly, Abdel-Moneim et al. [32] observed dose-dependent changes in MDA content and SOD activity following supplementation with curcumin nanoparticles, providing further evidence of the dose-dependent effects of curcumin on antioxidant status. Taken together, these findings suggest that the estimated supplementation range of approximately 139–188 mg/kg may provide a useful reference for improving intestinal antioxidant status in medium-growing broilers aged 57–84 d under the dietary and experimental conditions of the present study.

One limitation of this study is that the stability of β-actin was not independently validated for RT-qPCR normalization. Therefore, the findings regarding antioxidant-related gene expression should be interpreted with caution.

## 5. Conclusions

Dietary curcumin supplementation improved ileal morphology and intestinal antioxidant status in broilers aged 57–84 d. These improvements were accompanied by increased antioxidant enzyme activities and upregulated expression of antioxidant-related genes associated with Nrf2 signaling, suggesting its possible involvement in antioxidant defense. Based on the estimated breakpoints (approximately 139–188 mg/kg) and the overall antioxidant responses, dietary supplementation with approximately 188 mg/kg of active curcumin may be a suitable level for supporting intestinal antioxidant status under the experimental conditions of this study. These findings suggest that curcumin has potential as a natural feed additive to support intestinal health and antioxidant defense in broilers.

## Figures and Tables

**Table 1:** Composition and nutrient levels of the broiler complete diets (as-fed basis).

Item	Diets					
Ingredient, g/kg	Diet 1	Diet 2	Diet 3	Diet 4	Diet 5	Diet 6
Corn	686.20	686.20	686.20	686.20	686.20	686.20
Soybean meal	192.80	192.80	192.80	192.80	192.80	192.80
Corn gluten meal	47.85	47.85	47.85	47.85	47.85	47.85
soya-bean oil	32.50	32.50	32.50	32.50	32.50	32.50
CaHPO_4_ ^a^	6.50	6.50	6.50	6.50	6.50	6.50
CaCO_3_ ^a^	15.00	15.00	15.00	15.00	15.00	15.00
NaCl ^a^	3.00	3.00	3.00	3.00	3.00	3.00
L-lysine hydrochloride ^b^	1.20	1.20	1.20	1.20	1.20	1.20
DL-methionine ^b^	0.50	0.50	0.50	0.50	0.50	0.50
L-threonine ^b^	0.95	0.95	0.95	0.95	0.95	0.95
Micronutrients ^c^	3.50	3.50	3.50	3.50	3.50	3.50
Curcumin products ^d^	0.00	0.11	0.22	0.33	0.43	0.54
Corn starch ^d^	10.00	9.89	9.78	9.67	9.57	9.46
Nutrient levels, g/kg						
Metabolizable energy, kcal kg^−1 e^	3106	3106	3106	3106	3106	3106
Crude protein ^f^	160.95	160.71	161.01	160.89	160.67	160.73
Lysine ^e^	8.30	8.30	8.30	8.30	8.30	8.30
Methionine ^e^	3.50	3.50	3.50	3.50	3.50	3.50
L-threonine ^e^	5.80	5.80	5.80	5.80	5.80	5.80
Tryptophan ^e^	1.60	1.60	1.60	1.60	1.60	1.60
Methionine + Cysteine ^e^	5.60	5.60	5.60	5.60	5.60	5.60
Ca ^f^	6.98	7.08	6.98	7.04	6.97	7.05
Non-phytate P ^e^	2.50	2.50	2.50	2.50	2.50	2.50

^a^ Reagent grade. ^b^ Feed grade. ^c^ Provided per kilogram of diet: VA 10000 IU, VD_3_ 3750 IU, VE 27.5 IU, ′VK_3_ 2.5 mg, VB_1_ 2.5 mg, VB_2_ 8 mg, VB_6_ 3.75 mg, VB_12_ 0.025 mg, pantothenic acid calcium 12.5 mg, niacin 45 mg, folic acid 1.25 mg, biotin 0.125 mg, choline 750 mg, Cu (CuSO_4_·5H_2_O) 7 mg, Fe (FeSO_4_·H_2_O) 80 mg, Zn (ZnSO_4_·H_2_O) 555 mg, Mn (MnSO_4_·H_2_O) 660 mg, Se (Na_2_SeO_3_) 0.15 mg, I (Ca(IO_3_)_2_·H_2_O) 0.50 mg, and phytase 0.3 mg. ^d^ Curcumin products added in place of the equivalent weight of corn starch to produce treatment diets. ^e^ Calculated values. ^f^ Values determined by analysis, and each value is based on triplicate determinations.

**Table 2:** Primer sequences for real-time qPCR amplification *.

Genes	GenBank ID	Primer Sequences	Product Length (bp)
*CAT*	NM_0010311215.2	F: 5′-TTGCTGGAGAATCTGGGTC-3′	186
		R: 5′-CCTTCAAATGAGTCTGAGGGTT-3′	
*GPX1*	NM_001277853.2	F: 5′-ACGGCGCATCTTCCAAAG-3’	73
		R: 5′-TGTTCCCCCAACCATTTCTC-3′	
*GPX3*	NM_001163232.3	F: 5′-CAAACTGCTGCGGAGGC-3′	240
		R: 5′-AGGACTGTGAGCGGGTGTAG-3′	
*GPX4*	NM_001346448.1	F: 5′-CTTCGTCTGCATCATCACCAA-3′	72
		R: 5′-TCGACGAGCTGAGTGTAATTCAC-3′	
*Nrf2*	MN416129.1	F: 5′-GGCCACCCTAAAGCTCCATT-3′	177
		R: 5′-GGCTTCACTGAACTGCTCCT-3′	
*SOD*	XM_027454289.1	F: 5′-GACCTGCCCTACGACTATGG-3′	136
		R: 5′-TTGCCAGCGCCTCTTTGTA-3′	
*β-actin*	NM_205518.1	F: 5′-CAGCCATCTTTCTTGGGTAT-3′	169
		R: 5′-CTGTGATCTCCTTCTGCATCC-3′	

* *CAT*, catalase; *GPX*, glutathione peroxidase; Nrf2, nuclear factor erythroid 2-related factor 2; *SOD*, superoxide dismutase; F, forward; R, reverse.

## Data Availability

The original contributions presented in this study are included in the article. Further inquiries can be directed to the corresponding author.
